# Association Between Smoking and Back Pain in a Cross-Section of Adult Americans

**DOI:** 10.7759/cureus.806

**Published:** 2016-09-26

**Authors:** Bart N Green, Claire D Johnson, Jeff Snodgrass, Monica Smith, Andrew S Dunn

**Affiliations:** 1 Publications, National University of Health Sciences; 2 Department of Occupational Therapy, Milligan College; 3 Department of Occupational Therapy, School of Health Sciences at Walden University; 4 Research, Life Chiropractic College West; 5 Chiropractic, VA Western New York Health Care System

**Keywords:** smoking, back pain, prevalence

## Abstract

Purpose: Back pain is the leading cause of global years lived with disability. This cross-sectional study assessed if a greater exposure to smoking cigarettes was associated with a greater prevalence of back pain.

Methods: This study examined data from 34,525 United States adults from the 2012 National Health Interview Survey. Analyses assessed the difference in back pain prevalence among current smokers, former smokers, and never smokers and the number of cigarettes smoked between current smokers with and without back pain.

Results: Back pain prevalence was 28%. There was a significant association between back pain and smoking, *X*^2^ (2, 599, n = 34, 241) = 546.3, *p* < .001. Back pain increased with increased smoking exposure; back pain was present in 23.5% of never-smokers, 33.1% of former smokers, and 36.9% of current smokers. The number of cigarettes smoked per day for current daily smokers was higher for those with back pain (Md = 13) than those without back pain (Md = 10), *U* = 2701065, *z* = -3.70, *p* < .001, *r* = .05.

Conclusions: Our findings suggest that there may be a biological gradient associated with exposure to smoking cigarettes and back pain in adult Americans.

## Introduction

Back pain is highly prevalent and the leading cause of global years lived with disability [1]. Back pain is the most common chronic painful condition in Americans [2] and is associated with reduced quality of life, human suffering, and enormous financial and social burdens [3]. Approximately $34 billion in direct cost each year is attributed to treating back pain in the United States (US) [4]. Various factors have been investigated to reduce the prevalence of back pain, including physical activity, work environment, and injury prevention strategies. It has been suggested that smoking may be associated with back pain in theoretical models [5-9], basic science studies [10-12], and epidemiological research [7, 13-14].

Inconsistent measures of association are reported in the epidemiologic studies to date and investigators have either looked at different samples or data are reported from other countries [7, 13]. Previous reports have shown that smokers had a higher prevalence of back pain than former smokers and non-smokers, but the prevalence data varied by country [7], a small increased risk for back pain in smokers [15], and an increased risk of back pain in US adult smokers [14]. However, assessing the potential of a biological gradient between smoking and back pain has not been reported in US adults using data from the National Health Interview Survey (NHIS), a nationally representative cross-sectional epidemiologic survey.

Evaluation for the potential of an association between increasing levels of smoking frequency (dose) and the prevalence of back pain in US adults may help inform a better understanding of the possible interaction between smoking and back pain and to assist in designing population-based multivariable studies to assess for risk factors for back pain. Therefore, the purpose of this study was to identify if a greater exposure to smoking cigarettes was associated with a greater prevalence of back pain in a large sample representative of the current US adult population. The study hypothesis was that the prevalence of back pain would be greater in current smokers than in former smokers and never smokers. A secondary hypothesis was that current smokers who smoked more cigarettes would report a higher prevalence of back pain than those who smoked fewer cigarettes.

## Materials and methods

The Strengthening the Reporting of Observational Studies in Epidemiology (STROBE) statement [16], as it applies to cross-sectional studies, was used to prepare this report.

### Study design and data source

We performed a secondary cross-sectional analysis of US adults using data from the 2012 NHIS [17].

### Setting

NHIS is an annual interview that is done in-person between trained computer-assisted interviewers and the survey respondent and the methods are reported thoroughly elsewhere [17].  

### Participants and variables

The population for this sample was adult (age greater than or equal to 18 years) Americans and was estimated to be approximately 313,914,000 [18]. The Sample Adult questionnaire from the 2012 NHIS was used for this study. In this questionnaire, one adult from each family interviewed was randomly selected as the ‘sample adult’. Any adult was eligible to participate, except for those who were institutionalized or actively serving in the US military or unless he or she is physically or mentally unable to do so [19]. This study used publicly available and de-identified NHIS data. The research protocol was reviewed and approved prior to the commencement of data analysis by the Walden University Institutional Review Board, approval #11-04-14-0058716. Participants provided consent to participate to the interviewer for the original data collection conducted by the NHIS. This data is released to the public for future secondary analyses and requires no further participant consent.

Respondents were asked if they had back pain for an entire day or longer in the previous three months. Cases were defined as those who answered “yes”. Those who reported smoking at least 100 cigarettes in their lifetime were considered to be current smokers or former smokers and those who did not were considered to be never smokers. Differentiation between current smokers and former smokers was determined by the answering the question, “Do you NOW smoke cigarettes every day, some days, or not at all?” Responses were recoded into four categories of a current smoker (smoke daily or some days), former smoker, and never smoker. The number of cigarettes smoked per day was a continuous variable with a range from 0 to 99+.

### Bias

The risk of bias is present in every study and has been described as the degree to which a research report has answered the research question free from bias [20]. Hoy and colleagues identified 11 criteria to assess the risk of bias in prevalence studies, including items such as appropriate sampling, acceptable definitions of cases, rigorous data collection methods, and other items pertinent to internal and external validity [20]. Several attempts were made to minimize bias for this study. The sample size was representative of the US national population, as NHIS methodology employed a complex, randomized, and multistage sampling strategy leading to a high response rate and large sample, allowing for generalizability to the population. Typically, data for NHIS sample adults were collected directly from the subjects, which decreased the chance for bias. Cases were defined as those who answered that they had experienced back pain in the previous three months for an entire day or longer. All NHIS interviewers were trained in a standardized manner and the interview was conducted with computer assistance to decrease variation in how questions were asked.

### Sample size

Using G*Power 3.1.9.2 for Windows (Universität Düsseldorf, Germany) [21], a power analysis was performed [22]. A medium effect size of  0.3 was selected, with alpha at 0.05 (2-tailed) and power of 0.95, based upon the methods of Faul and colleagues [23]. The estimated number of subjects required for the Chi-square and Mann-Whitney U were 146 and 220, respectively.

### Statistical analysis

All assumptions necessary to use the selected tests were met. Chi-square test for independence with weighting for population estimates was used to test the association between smoking and back pain. The Mann-Whitney U test was used to assess for a difference in the median number of cigarettes smoked per day between current daily smokers with and without back pain, current some-day smokers with and without back pain, and all current smokers (daily and some days) with and without back pain. Cases with missing data were excluded from the analysis. We used SPSS Complex Samples Analysis, version 21 (IBM Inc, Armonk, NY) for statistical testing.

## Results

### Participants

There were 34,525 total respondents (79.7% response) and 284 cases (0.8%) had missing data and were excluded from the statistical analysis, yielding a final sample of 34,241.  

### Descriptive data

Back pain was present in 10,078 cases (28%). Most people had never smoked, with 6,436 current smokers in the sample (prevalence = 18%). Descriptive statistics are presented in Table 1.

**Table 1:** Descriptive Statistics for Sample and Population Estimates 95% CI = 95% confidence interval

Variable	Unweighted Count	%	Population Estimate	Standard Error	95% CI Lower Bound	95% CI Upper Bound
Back Pain						
Yes	10,078	28.0	65,823,057	965,737	63,922,579	67,723,534
No	24,427	72.0	168,937,180	1,844,349	165,307,679	172,566,680
Total	34,505	100.0	234,760,237	2,291,291	230,251,198	239,269,275
Smoking						
Current	6,436	18.1	42,098,139	813,046	40,498,141	43,698,136
Former	7,584	22.2	51,621,850	865,475	49,918,678	53,325,021
Never	20,236	59.8	139,327,445	1,617,264	136,144,825	142,510,064
Total	34,256	100.0	233,047,434	2,287,231	228,546,384	237,548,483
Sex						
Male	15,273	44.2	113,070,897	1,471,455	110,175,215	115,966,578
Female	19,252	55.8	121,849,773	1,395,324	119,103,909	124,595,636
Total	34,525	100.0	234,920,670	2,292,625	230,409,005	239,432,334
Race						
Hispanic	5,859	14.9	34,946,432	816,213	33,340,203	36,552,660
Non-Hispanic White	20,842	67.2	157,787,860	2,071,449	153,711,448	161,864,271
Non-Hispanic Black	5,282	11.9	27,885,414	626,598	26,652,329	29,118,498
Non-Hispanic Asian	2,168	5.3	12,415,030	424,711	11,579,238	13,250,821
Non-Hispanic All Other	372	0.8	1,885,934	198,313	1,495,671	2,276,196
Total	34,525	100.0	234,920,670	2,292,625	230,409,005	239,432,334

### Main results

**Figure 1:**
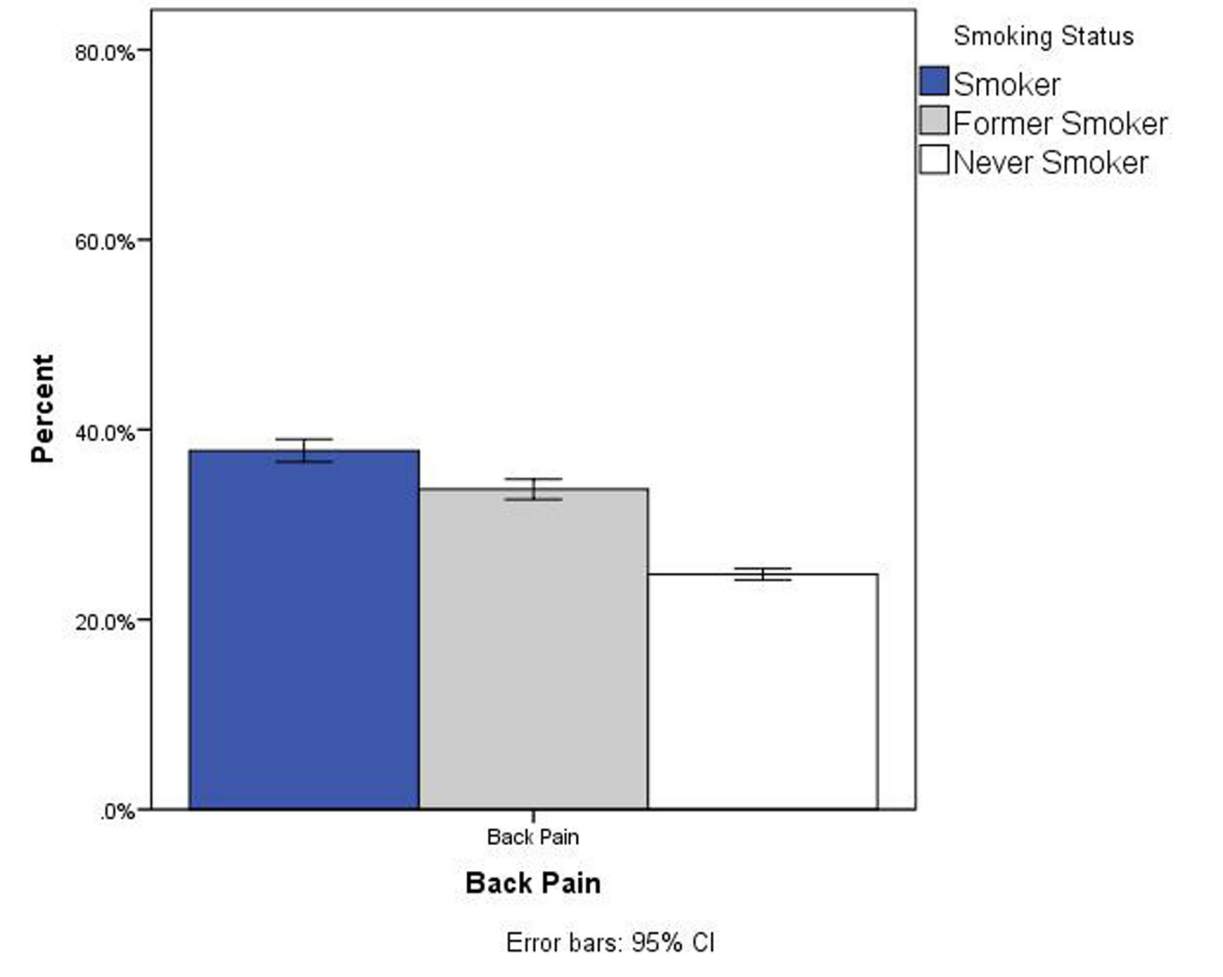
Prevalence of Back Pain by Smoking Status Back pain was estimated to be present in 23.5% of never-smokers (95% CI, 22.7 - 24.3), 33.1% of former smokers (95% CI, 31.8 - 34.4), and 36.9% of current smokers (95% CI, 35.3 - 38.4)

**Figure 2:**
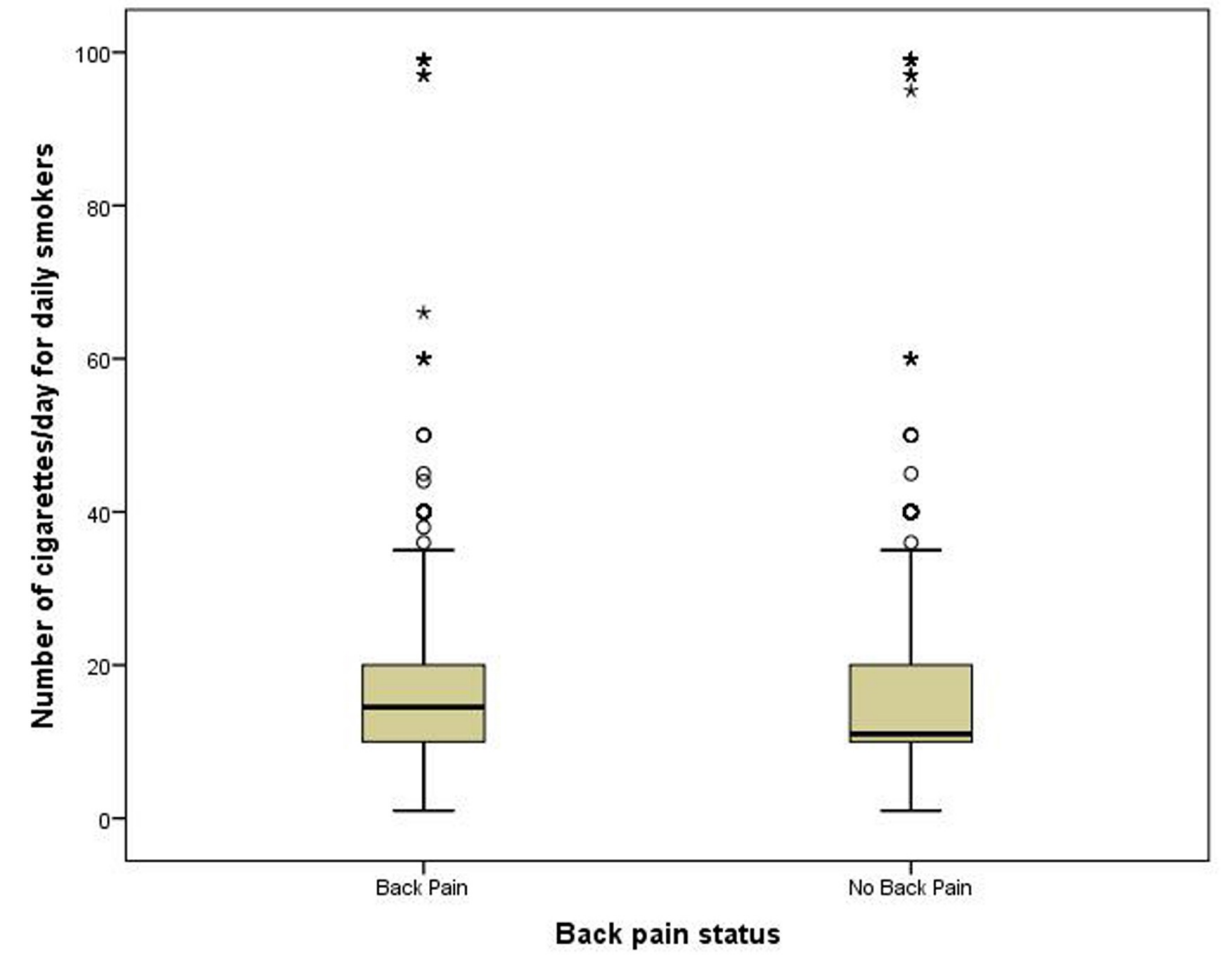
Box Plot of Median Number of Cigarettes Smoked for Daily Smokers with and without Back Pain The median number of cigarettes smoked daily for people without back pain was 10 and for those with back pain it was 13.

A significant association was found between back pain and smoking status, *X*^2^ (2,599, n = 34, 241) = 546.3, *p* < .001. As shown in Figure 1, back pain was less prevalent in never smokers than former smokers and both of these groups had less back pain prevalence than current smokers. For current daily smokers, the Mann-Whitney U test revealed a significant difference in the number of cigarettes smoked per day of those with back (n = 1,915) pain and those without back pain (n = 3005), *U* = 2,701,065, *z* = -3.70, *p* < .001, *r* = .05 (Figure 2). For current some-day smokers, there was no difference in the number of cigarettes smoked per day of those with back pain (Md = 3, n = 485) and those without back pain (Md = 3, n = 919). When combining all smokers (daily and some-day smokers), there was no difference in the number of cigarettes smoked per day of those with back pain (Md = 10, n = 2400) and those without back pain (Md = 10, n = 3924).

## Discussion

### Key results

This study found that current smokers had a higher prevalence of back pain than former smokers and never smokers and that former smokers had a higher prevalence of back pain than never smokers. Among current daily smokers, those who smoked more cigarettes per day had a statistically higher prevalence of back pain. This suggests that more frequent exposure to smoking cigarettes may possibly be associated with a higher prevalence of back pain.

### Interpretation

Our findings support and add to the knowledge generated by previous US studies. One meta-analysis suggested that smokers had a higher prevalence of back pain than former smokers and non-smokers and that former smokers had a higher prevalence of back pain than non-smokers and that prevalence data varied by country and that measures of association were inconsistent across geographic regions [7]. A study of 1976-1980 data from the National Health and Nutrition Examination Survey II (NHANES II) showed a small increased risk for back pain in smokers compared to non-smokers (RR = 1.13) with a higher prevalence of back pain among the heaviest smokers (three or more packs per day) when compared to non-smokers [15]. In this study by Deyo and Bass, the prevalence of low back pain was 10.9% versus 9.6% among those who never smoked. In their study, the one-year prevalence of back pain increased as the greatest amount of smoking was considered, showing a 9.6% prevalence for non-smokers versus 25.1% for smokers who smoked more than three packs per day [15]. While the data from Deyo and Bass are nearly four decades old and complex samples analysis was not used to address intended variations in sampling the population, they were the first US data to show a potential biological gradient between smoking and back pain. Data from the current study, which used complex samples analysis, support this trend and showed that the prevalence of back pain among Americans was higher with the prevalence of back pain rising from 23.5% for never smokers to 33.1% in former smokers and 36.9% in current smokers. The current study also supports the hypothesis that heavier daily smokers had a higher prevalence of back pain than lighter daily smokers. However, in current some-day smokers, there was no difference in the number of cigarettes smoked per day of those with back pain and those without back pain, which challenges the hypothesis or suggests some other variables are more influential in some-day smokers.

A later US study of data from the 2002 NHIS showed that US adults with low back pain or neck pain had riskier health behaviors than adults without back or neck pain and that the odds of back pain were higher in smokers (OR = 1.3); however, this study did not assess whether there may be a dose-response relationship between smoking levels and back pain [14]. Our data supports and provides an update of NHIS data and provides new information pertaining to the increased prevalence of back pain in NHIS respondents who are daily smokers that smoke more cigarettes.

There are plausible considerations that smoking may have a dose-response relationship with back pain. Biological mechanisms have been hypothesized for the relationship between smoking and back pain and range from neurological to biomechanical mechanisms. It has been suggested that nicotine, through its excitatory effects, alters the perception and threshold for pain, increasing the self-reporting of pain [6]. Thus, greater exposure to nicotine may increase the amount of pain perception. Smoking also increases the level of circulating pro-inflammatory cytokines, which signal the central nervous system and may lead to amplification of pain [7]. It has also been suggested that smoking increases inflammation [24]. Frymoyer, et al. [25] were the first to propose that people with back pain had a greater prevalence of coughing and that there was a “… possibility that mechanical stresses induced by coughing may be relevant to the low-back complaint.” However, this mechanism was not fully supported by Deyo and Bass who used multivariate analysis to control for chronic cough symptoms and found that this did not eliminate the association of smoking with back pain [15]. Other plausible biological mechanisms have been proposed as an underlying basis of an association between smoking and back pain. While this is not an exhaustive review of all possible explanations for this relationship, it does show biological plausibility, one of the criteria necessary when discussing possible dose-response relationship and epidemiological studies of association [26].

### Limitations

This exploratory study only investigated smoking and low back pain; other biopsychosocial factors that may relate to the amount of smoking or low back pain were not explored. It is possible that some of these variables influenced the prevalence of back pain. However, considering that covariables were likely distributed across this large and complex sample design, we feel that the findings of this study suggest the presence of a biological gradient or dose-response relationship between smoking and back pain. This study is also limited by the survey data collection and the data are based on self-report and may be influenced by recall bias. One of the inherent limitations to the study is the vague definition of the survey relating to the term “back pain”, which could have influenced the prevalence of back pain in the sample. This is a cross-sectional research design and, therefore, cannot imply causation of back pain from smoking [27]. The survey was limited to non-institutionalized and non-military adults. Considering the psychological and physical demands upon these populations, it is possible that the estimates provided from this research underreport the prevalence of back pain and the magnitudes of association of some of the independent variables with back pain. This study only investigated adults and, therefore, did not analyze smoking in children and teens. Thus, the results of the present study may not be applicable to other populations.

### Generalizability

The present study provides robust data on a large and representative sample of US adults. There is a higher prevalence of back pain among smokers than former smokers and never smokers and a higher prevalence of back pain among those who smoke more cigarettes. Given the large sample size obtained from independent sampling using a complex sampling strategy and weighting to population estimates using SPSS Complex Samples Analysis, it is felt that the results of this study are generalizable to adults living in the US.

## Conclusions

Further attention is needed to clarify smoking as a potential risk factor associated with the substantial financial and social burden of back pain. The present study found that current smokers who smoked more cigarettes per day had a higher prevalence of back pain. These findings suggest that there might be a biological gradient associated with exposure to smoking cigarettes and back pain in US adults. The risk factor of smoking should be considered as one of many factors when developing research studies or intervention strategies for those with back pain.  
